# Supplementary material for: Mass spectrometry imaging-based explainable machine learning reveals the biochemical landscapes of the mouse brain
Source: Free Neuropathol. 2026 Apr 28;7:9. doi: 10.17879/freeneuropathology-2026-9413 (PMC13129809; doi:10.17879/freeneuropathology-2026-9413)
Supplement: Supplementary file 1 [file freeneuropathol-07-09-9413-s1.pdf]

Supplementary material

- **Supplementary Figures S1–S3**
- **Supplementary Tables S1–S3**

Supplementary Figure S1: Comparison of m/z selection strategies for plaque-associated signals

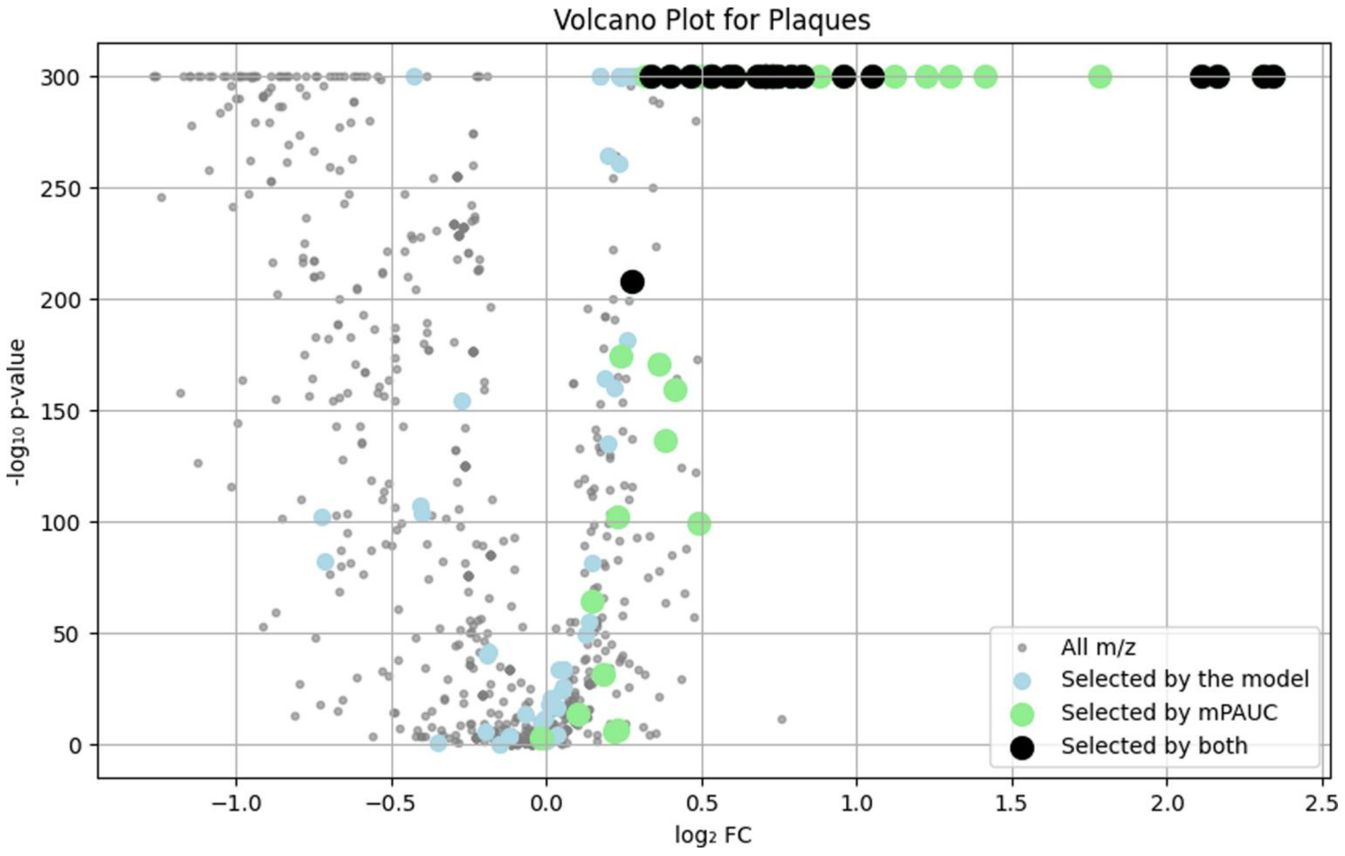

Fig. S1 Different m/z selection strategies applied to the Aβ plaque (PLQ) category. The model-based approach also selects m/z values that are shared with other categories and can therefore have a negative log_2 fold-change. The mPAUC method is selective for *m/z* values that are specific to PLQ because it compares values pairwise. The intersection of both methods results in highly selective masses for PLQ, while their union may help gather all potential m/z values associated with PLQ.

Copyright: © 2026 The author(s). This is an open access article distributed under the terms of the Creative Commons Attribution 4.0 International License (https://creativecommons.org/licenses/by/4.0/), which permits unrestricted use, distribution, and reproduction in any medium, provided the original author and source are credited, a link to the Creative Commons license is provided, and any changes are indicated. The Creative Commons Public Domain Dedication waiver (https://creativecommons.org/publicdomain/zero/1.0/) applies to the data made available in this article, unless otherwise stated.

Supplementary Figure S2: Model-explanation view of CBLA category weights

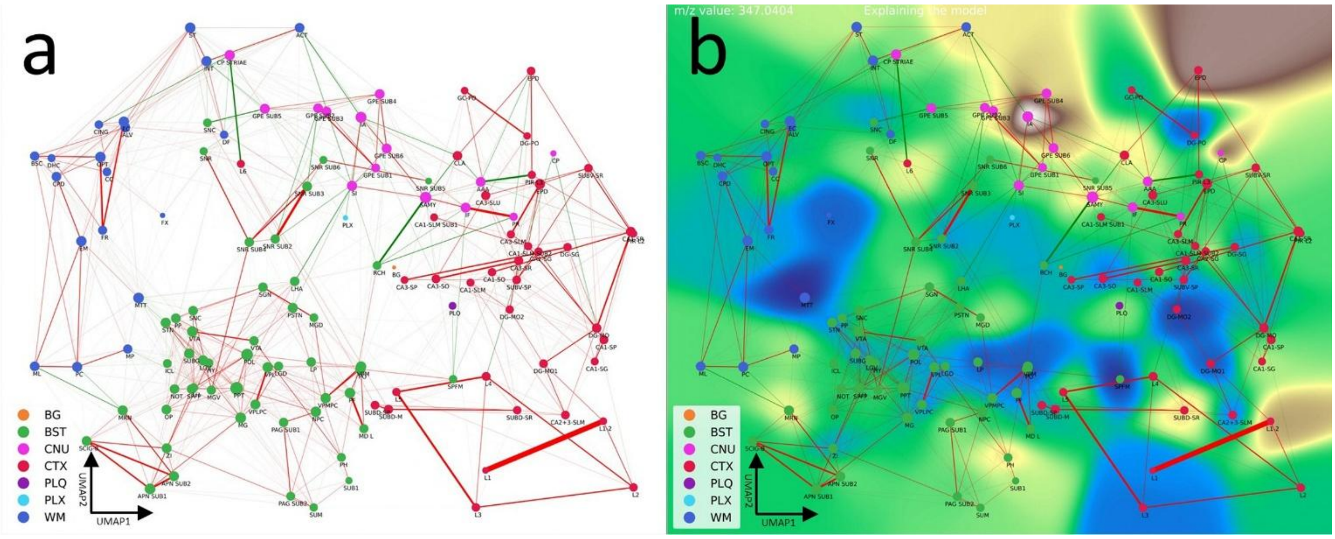

Fig. S2 Explaining the model. a, Graph of the CBLA used for explaining the model weights, where we used a Bayesian logistic regression model for classifying the categories and using the category weights for each node before applying dimensionality reduction. **b**, VLV for explaining the model weights. Here, the nodes represent the learned model weights for each category. The model weight coefficients for the m/z 347.0404 are overlaid with the ’terrain’ Matplotlib color scheme. Notice that, like for the data VLV (**Fig. 4**), the IA and EPD categories show a strong response, confirming the model has found this m/z predictive for these categories. However, unlike the data VLV, the model does not show a strong signal for the CA2-sg or the L1–2 categories, potentially showing that the model could be improved. Legend: BG, background; BST, brainstem; CNU, cerebral nuclei; CTX, cerebral cortex; PLQ, Aβ plaques; PLX, choroid plexus; WM, white matter.

freeneuropathology.org

Copyright: © 2026 The author(s). This is an open access article distributed under the terms of the Creative Commons Attribution 4.0 International License (https://creativecommons.org/licenses/by/4.0/), which permits unrestricted use, distribution, and reproduction in any medium, provided the original author and source are credited, a link to the Creative Commons license is provided, and any changes are indicated. The Creative Commons Public Domain Dedication waiver (https://creativecommons.org/publicdomain/zero/1.0/) applies to the data made available in this article, unless otherwise stated.

Supplementary Figure S3: UMAP visualization of all annotations

The UMAP visualizations of the *m/z* content of the annotations are given in the Extended Data, **Fig. S3**, showing that different brain regions have distinct lipidomic profiles, enabling their visualization and annotation.

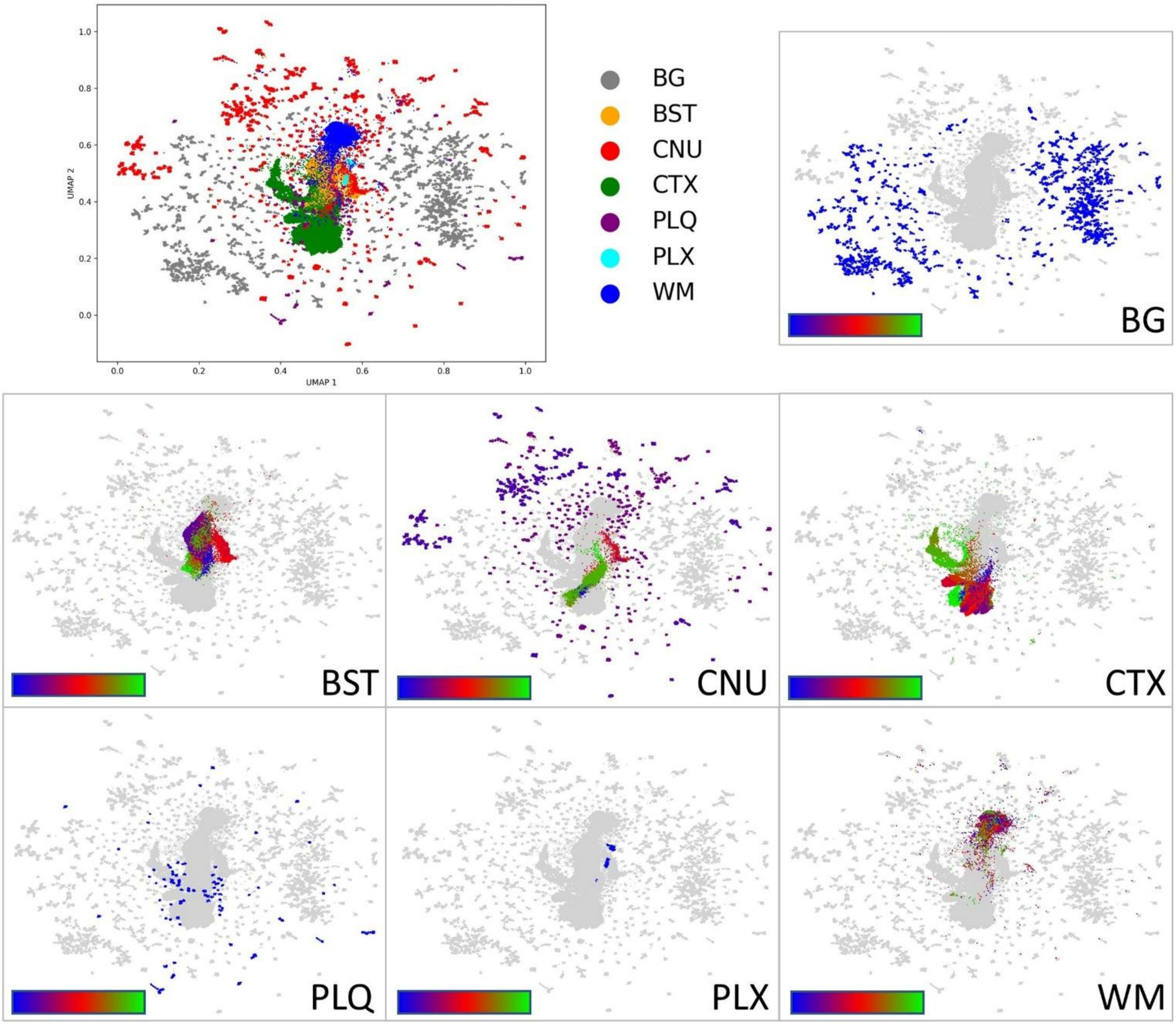

Fig. S3 Analyzing lipid profiles of brain regions. The high-dimensional MSI data was reduced to two dimensions using UMAP [16]. The 123 regions were grouped into the main categories according to their anatomical locations [9] or designated as background (BG; ground truth). Each main category was also colorized with a BRG color scheme, showing the diversity within the categories. This visualization shows that annotated points from the same categories tend to distribute similarly in the low-dimensional visualization, which would be expected if the annotations were correct. On the other hand, our annotations capture extreme diversity in the data, especially in the CNU, WM, CTX, and BST categories. Legend: BG, background; BST, brainstem; CNU, cerebral nuclei; CTX, cerebral cortex; PLQ, Aβ plaques; PLX, choroid plexus; WM, white matter.

freeneuropathology.org

Copyright: © 2026 The author(s). This is an open access article distributed under the terms of the Creative Commons Attribution 4.0 International License (https://creativecommons.org/licenses/by/4.0/), which permits unrestricted use, distribution, and reproduction in any medium, provided the original author and source are credited, a link to the Creative Commons license is provided, and any changes are indicated. The Creative Commons Public Domain Dedication waiver (https://creativecommons.org/publicdomain/zero/1.0/) applies to the data made available in this article, unless otherwise stated.

Supplementary Table S1–S2

- Supplementary Table S1: model-based mapping (CSV file, 425KB)
- Supplementary Tables S2: mPAUC mapping (CSV file, 51KB)

Supplementary Table S3: Identification of selected *m/z* features

m/z	Putative annota-tion	Associated regions
347.0404	IMP	CP, GPE, EPD, SAMY_IA, SAMY_AAA, CA2-sg, CLA
480.3097	LysoPE(18:0)	CTX_L1
540.0534	cyclic ADP-ribose	CTX_L1–L5, PIR
722.5119	PE O-36:5	SNR, SNC, GPE, CP, CA3, PIR
795.5195	PG 38:5	SUM, PLQ, DG, CA1, CA2
807.5005	PI(32:1) class	DG-mo, HIP
857.5171	PI(36:4) class	CTX_L1–L5, HIP, PIR
865.5031	PI/PG (unresolved)	CA1, DG, CA2, CA3
1179.7308	GM3(36:1)	PLQ, CA1, CA2-slm/so, CA2, CA3, DG, DG-mo

Table S3. Putative identification and spatial localization of selected m/z features. Putative lipid annotations were assigned as described in Methods (see Putative lipid annotation), and are reported here as hypothesis-generating assignments requiring orthogonal validation (e.g., MS/MS).

Abbreviations:

AAA - anterior amygdalar area,

ADP - adenosine diphosphate,

CA1 - cornu ammonis area 1,

CA2 - cornu ammonis area 2,

CA2-sg - cornu ammonis area 2, stratum granulosum,

CA2-slm/so - cornu ammonis area 2, stratum lacunosum-moleculare/stratum oriens,

CA3 - cornu ammonis area 3,

CLA - claustrum,

CP - caudate putamen,

CTX_L1 - cerebral cortex layer 1,

CTX_L1–L5 - cerebral cortex layers 1 to 5,

DG - dentate gyrus,

DG-mo - dentate gyrus, molecular layer,

EPD - endopiriform nucleus, dorsal part,

GM3 - monosialodihexosylganglioside (ganglioside GM3),

GPE - globus pallidus, pars externa,

HIP - hippocampus,

IA - intercalated amygdaloid nuclei,

IMP - inosine monophosphate,

PE - phosphatidylethanolamine,

PG - phosphatidylglycerol,

PI - phosphatidylinositol,

PIR - piriform cortex,

PLQ - Aβ plaque category,

SAMY - striatum-like amygdalar nuclei,

SNC - substantia nigra, pars compacta,

SNR - substantia nigra, pars reticulata,

SUM - supramammillary nucleus

Copyright: © 2026 The author(s). This is an open access article distributed under the terms of the Creative Commons Attribution 4.0 International License (https://creativecommons.org/licenses/by/4.0/), which permits unrestricted use, distribution, and reproduction in any medium, provided the original author and source are credited, a link to the Creative Commons license is provided, and any changes are indicated. The Creative Commons Public Domain Dedication waiver (https://creativecommons.org/publicdomain/zero/1.0/) applies to the data made available in this article, unless otherwise stated.
